# Novel mapping methods to describe utilization of free breast cancer screening from a state program

**DOI:** 10.1016/j.pmedr.2021.101415

**Published:** 2021-05-29

**Authors:** Kelly D. Hughes, David Haynes, Anne M. Joseph

**Affiliations:** a Minnesota Department of Health, Sage Program, 85 7th Place E, St. Paul, MN 55101 USA; b University of Minnesota, Institute for Health Informatics, Suite 8-100, 516 Delaware Street SE, Minneapolis, MN 55455 USA; c University of Minnesota, Department of Medicine, Division of General Internal Medicine, 420 Delaware St SE, MMC 194, Minneapolis, MN 55455 USA

**Keywords:** Geographic Mapping, Geographic Information Systems, Female, Humans, Breast Neoplasms, Early Detection of Cancer, Program Evaluation, Medically Uninsured, Minnesota

## Abstract

**Introduction:**

The National Breast and Cervical Cancer Early Detection Program (NBCCEDP) is a cancer screening program whose mission is to reduce cancer morbidities for uninsured and underinsured women. A primary activity is to connect women to breast cancer screening. The eligible population and utilization of NBCCEDP screening services have never been quantified at a sub-state level, which hampers effective program evaluation. Here, the Minnesota NBCCEDP, “Sage”, serves as a case study to demonstrate novel spatial analysis methods that depict variation of screening rates at the local level.

**Methods:**

Women who received breast cancer screening through Sage between 2011 and 2015 were geocoded (N = 74,712 screenings); analysis occurred between 2017 and 2019. We determine an eligible population using a synthetic population dataset that provides geographic residence and demographic information. We introduce a novel spatial analysis technique, spatially adaptive filters (SAFs), to create a utilization map of Sage breast screening services by Minnesota women.

**Results:**

Between 2011 and 2015, an average of 36,979 women per year were eligible for NBCCEDP breast cancer screening services, representing 3% of the Minnesota female population 40 and older. For Minnesota NBCCEDP eligible women, the state average breast cancer screening utilization rate was 37.2%, but varied considerably by local regions within the state (range 0% to 131%, SD = 18.7%).

**Conclusions:**

This geospatial model estimated screening service utilization at the local level and enables Minnesota’s Sage program to target specific areas they have yet to reach. Similar programs could employ this model to direct program activities.

## 1 Introduction

Cancer is the second leading cause of mortality among women in the United States, and 30% of new cancer diagnoses in 2020 among women will be breast cancers (Hahn et al., 2018, American Cancer Society, 2020). Breast cancer mortality can be reduced via early detection through screening, yet disparities in breast cancer screening persist. Nationally, low-income women (<$15,000/year) ages 50 to 64 are less likely to be screened than women with higher income (>$50,000/year), 71.9% compared to 83.0% (Centers for Disease Control and Prevention, 2018). Women without health insurance are screened at a lower rate (54.7%) than women with insurance (80.5%) (Centers for Disease Control and Prevention, 2018). Racial disparities in screening also exist, as Asian and American Indian women are screened at lower rates than other groups; women of color and American Indians face disproportionate barriers to screening, such as having lower rates of access to health care (Artiga et al., 2016, Kaiser Family Foundation, 2018). Recognizing that access to early breast cancer detection is a health priority among low-income and uninsured women, the National Breast and Cervical Cancer Early Detection Program (NBCCEDP) was established in 1991 to provide screening services for such populations.

NBCCEDPs are present in every US state and territory, and in some tribal areas (Tangka et al., 2006). To evaluate NBCCEDP delivery of screening services, previous research sought first to quantify the eligible population and uptake of services, often at the national level. Among US states, Tangka and colleagues estimated that 4 million women 40 to 64 years of age were eligible for NBCCEDP breast screening services, and 13.2% received screening in 2002–2003 (Tangka et al., 2006). Howard and colleagues updated this work using 2011–2012 data and estimated a larger eligible population of 5 million women 40 to 64, with an average national screening utilization of 10.6% (Howard et al., 2015). A final study considering years 2006 to 2010 placed the NBCCEDP national breast screening utilization rate at 19.9% (Subramanian et al., 2015). Of these papers, only one reported state-level screening rates, and although screening rates for all states were reported, these were not labeled and so the specific screening rate for any state was not identifiable (Howard et al., 2015). To better inform program effectiveness, NBCCEDPs would benefit from spatially generated state and local estimates of the eligible population and service utilization.

Variation in utilization of services within NBCCEDP jurisdictions is currently unknown. Local variation in uptake of health services is critical to addressing disparities, as health and disease are linked to demographic characteristics of a local population and resources in their immediate environment (Gibson et al., 2002, Dummer, 2008). That is, local mapping reveals the story of health and disease (Koch, 2005). Eligible population uptake of NBCCEDP screening services may vary considerably. Howard and colleagues found that NBCCEDP breast screening utilization ranged from 3.2% to 52.8% among US states in 2011 to 2012 (however, specific states were not labeled) (Howard et al., 2015). Similar variation describing the uptake of services for NBCCEDPs might be found below the state level, but to date there are no peer-reviewed accounts of this information. There is variation in breast cancer screening utilization in the general population. The 500 Cities Project, which creates small area estimates for select US cities from national datasets such as Behavioral Risk Factor Surveillance System (BRFSS), shows a range of 60% to 83.5% for biannual breast cancer screening utilization among general population women in large US cities, and similar ranges of variation at the neighborhood level (Cebters for Disease Control and Prevention, National Center for Chronic Disease Prevention and Health Promotion, Division of Population Health, 2020). Screening utilization varies within the states, as cancer screening does not reach all of the intended population (Earp et al., 1995, Coughlin et al., 2008).

New geospatial techniques are needed to generate the high-resolution yet high-accuracy data needed to evaluate NBCCEDP effectiveness and disparities in program use. We demonstrate the use of spatially adaptive filters (SAFs) and a synthetic population dataset developed by the Research Triangular Institute (RTI) to calculate a breast cancer screening eligible population, and variation in utilization rates at the neighborhood level for the Minnesota NBCCEDP, “Sage”. SAFs have been used to describe cancer incidence and mortality patterns, but have never been applied to programmatic data (Beyer and Rushton, 2009, Tiwari and Rushton, 2005). For Minnesota, the implementation of these methods will improve understanding of the uptake of program services and allow for targeting of specific locations where Sage can deploy limited resources. In a wider scope, this project demonstrates the utility of accurate sub-state estimates of target populations, by documenting obscured local variation in utilization of services.

## 2 Methods

### 2.1 Program description

Sage is the NBCCEDP of Minnesota, whose primary activities are program recruitment, client connection to resources through patient navigation, creating a network of resources that provide access to cancer-related healthcare, and paying for breast and cervical screening and diagnostic services. The entire state of Minnesota is Sage’s service catchment area; Sage partners with over 400 clinics across the state, and serves women residing in every county in the state of Minnesota. Previous work estimated a Sage eligible population of 35,000 (Howard et al., 2015). Sage screens approximately 15,000 uninsured and underinsured low-income women annually (determined by Sage’s database of every woman screened since inception, 1991). Minnesota has one of the highest rates of health insurance access in the nation, yet the state experiences persistent disparities in breast cancer screening, incidence, and mortality that reflect national averages, as well as similar race and ethnicity disparities in income and health care access (Centers for Disease Control and Prevention, 2018, American Cancer Society, 2017). This project performs secondary analysis of observational data for the purpose of program evaluation, and IRB review was not required. Sage screening activity from 7/1/2010 to 6/30/2015 (the fiscal year starts July 1st and ends June 30th) was analyzed. During this time period, Sage conducted recruitment of women previously served by the program and women never reached by the program, partnered with clinics to engage local women for screening and conduct in-clinic recruitment, conducted broad recruitment campaigns by radio, newspaper, billboard, television and other means, and women were directed to the program through the American Cancer Society and National Breast Cancer Awareness Month, among many other grassroots organizations. The analysis was completed between the years 2017 and 2019.

Sage endorsed annual screening for average risk women, starting at age 40, with no prescribed end date; the end of regular screening was to be discussed between a woman and her physician and Sage supported screening as long as a woman pursued it. The majority of screenings are mammograms, but women may receive tomosynthesis or ultrasounds with doctor’s prescription.

### 2.2 Numerator

Sage eligibility and utilization rates were estimated based upon Sage’s breast cancer screening eligibility guidelines between 2010 and 2015. To qualify for services a woman must have had an income below 250% of the federal poverty level and be uninsured or underinsured, defined as any out-of-pocket costs for screening. Note, that each state NBCCEDP can set its own guidelines for eligibility, patterned after nationally recommended guidelines (NBCCEDP, 2020). For this analysis, Sage screening cases were defined as all instances of women 40 and older screened for breast cancer through Sage between the dates of analysis, which was 74,712 instances of screening, pulled from Sage’s database. Sage NBCCEDP federal funding is supplemented by state and non-profit funds and grants that contribute 20% to 25% of Sage’s total budget. Screening supported by all funding streams was included in the analysis and funding from non-federal sources was not conditional or allocated for a target population so that all funding sources serve all Sage eligible women. Additional tests, such as diagnostic mammograms that occurred after the initial screening, were not included as screenings in the analysis. Sage clients that were non-Minnesota residents were removed.

We used the Minnesota Department of Health geocoder to determine geographic coordinates from client addresses. We defined geocoded clients as those with an address match score ≥60; the lower bound of 60 was acceptable as we retroactively geocoded the data which affects match score quality (Goldberg et al., 2013). The address match score has a range 0 to 100, and is a result of the accuracy of the given address with the reference dataset of roads (Goldberg, 2011). In the case of multiple address matches we accepted the address with the highest score. Most screening instances (73%) had a geocode score of 80 or greater.

Some Sage clients did not have sufficient address information for geocoding, yet all clients did supply a zip code. When full address information is missing, it is common practice to geocode individuals to a zip code using zip code tabulated areas (ZCTA) (Rushton et al., 2006). We developed an algorithm in PostgreSQL v 10 that equally distributed all clients within a given ZCTA, if that client did not have a full address; this assigned point within the ZCTA served as the geocoded location of the screened client. PostgreSQL is open source software.

### 2.3 Denominator

The Research Triangular Institute (RTI) 2010 U.S. Synthesized Population dataset was used to calculate our eligible population (Wheaton et al., 2009). The synthetic dataset is a statistical model based on American Community Survey Public Use Microdata. It provides geographic coordinates and household characteristics (i.e., household size and income) for every synthetic household. The household record can be linked to a secondary statistically modeled person dataset. By linking the individual to the household, we created a geographic location for every statistically modeled woman in Minnesota.

We defined the potential eligible client population using synthetic population characteristics of age and income, reflecting Sage eligibility criteria (Eq. (1)). Additionally, age-appropriate American Indian or Alaska Native women screened through Indian Health Service were eligible for Sage regardless of income (Eq. (2)). The income threshold of < 250% federal poverty level was calculated through household income and size (Poverty Guidelines. ASPE, 2021). For women who were not American Indian or Alaska Native, we applied estimated rates of uninsured and underinsured to the potential eligible client population. From published public data, the underinsured rate in Minnesota was estimated to be 10%, and the uninsured rate for Minnesota women ages 40 to 64 with income <= 250 FPL was estimated at 12.6% (Schoen et al., 2011, United States Census Bureau. SAHIE, 2017). Together, this gives a proportion of 0.226 women who were uninsured or underinsured, which we applied to all women in Eq. (1).

*Equation* (1)*, income and insurance status based eligibility criteria*

(1) $$\mathrm{Eligible}Person\times 0.226=\left(Income-30350-\left(10800\times Householdsize\right)\leq 0\right)\;AND\;(sex=Women)\;AND\;(Age\;\geq 40)\;AND\;race\;NOT\;IN\;\left(American\;Indian,\;Alaska\;Native\right)$$

*Equation* (2)*, Indian Health Services eligibility criteria*

(2) EligiblePerson=(sex=Women)AND(Age≥40)ANDraceIN(AmericanIndian,AlaskaNative)

These two equations are used together to define all individuals whose characteristics meet the criteria for eligibility in the RTI dataset. This population of women was multiplied by five to generate a denominator for five years’ of Sage screening data, as women were considered eligible for screening every year.

### 2.4 Geospatial model

Sage breast cancer screening utilization rates were generated using the numerator and denominator populations in conjunction with spatially adaptive filters (SAFs), an advanced geospatial technique that creates stable rates by controlling for changes in population density (Beyer and Rushton, 2009, Tiwari and Rushton, 2005). The SAFs approach was selected for several reasons. SAFs do not rely on pre-defined administrative boundaries (i.e., zip codes, county), and are robust to issues that arise from working with small numbers, such as unstable estimates, suppression of data, and the Modifiable Areal Unit Problem (Fotheringham and Wong, 1991).

When applying SAFs, a dense grid is first “laid” over the area to be mapped (i.e., all of Minnesota); in our model we selected a grid of 9,522 points that were 5,000 m apart. 5,000 m is the smallest resolution possible with our current computation limitations; this grid size has been previously used successfully in SAF estimation approaches. (Yiannakoulias et al.) Next, centered at each grid point, the circle is expanded until it captures 500 individuals in the denominator (i.e., the Sage eligible population). When the circle’s area has been determined, a point utilization rate at the centered grid point is calculated; the numerator consists of all the Sage screened geocoded individuals within that same circle. The method for computing the areas of each circle is complex and necessitated the big data platform Apache Spark, using the geospatial library GeoSpark; Apache Spark is open source software (Yu et al., 2019).

### 2.5 Utilization map

After point estimates were determined for the entire grid, an inverse distance weighted interpolation was applied across the Minnesota grid to calculate utilization rates between points, increasing the resolution of our map. The interpolation results in a smoothed surface with 873,983 cells, with a spatial resolution of 500 m^2^. The interpolation and final plotting of the map was accomplished with ArcPro v 2.4.

## 3 Results

All Sage clients screened for breast cancer between fiscal years 2011 and 2015 were used in the mapping process; 90% were geocoded to physical addresses, and the remaining 10% were assigned a location within a ZCTA using our algorithm. Sage clients self-identified as white (53%), Hispanic (22%), African American (9%), American Indian (7%), and Asian (3%). Six percent of clients did not provide racial information or were multi-racial. Almost half of all Sage clients, 48.9%, lived within the seven-county metro area of Minneapolis and St. Paul.

Our approach estimates that 36,979 Minnesota women 40 years and older were eligible for Sage breast cancer screening services per year between fiscal years 2011 and 2015, on average. This represents 3% of the Minnesota population of women 40 and older in 2010. The average breast screening utilization for Sage services, which assumes annual screening, was 37.2%. Utilization rates varied locally. The range of point estimates of utilization, for the spatially adaptive filters was 0% to 131%, with a standard deviation of 18.7%. Interpolated areas > 100% utilization accounted for 0.8% of all areas.

**Fig. 1:**
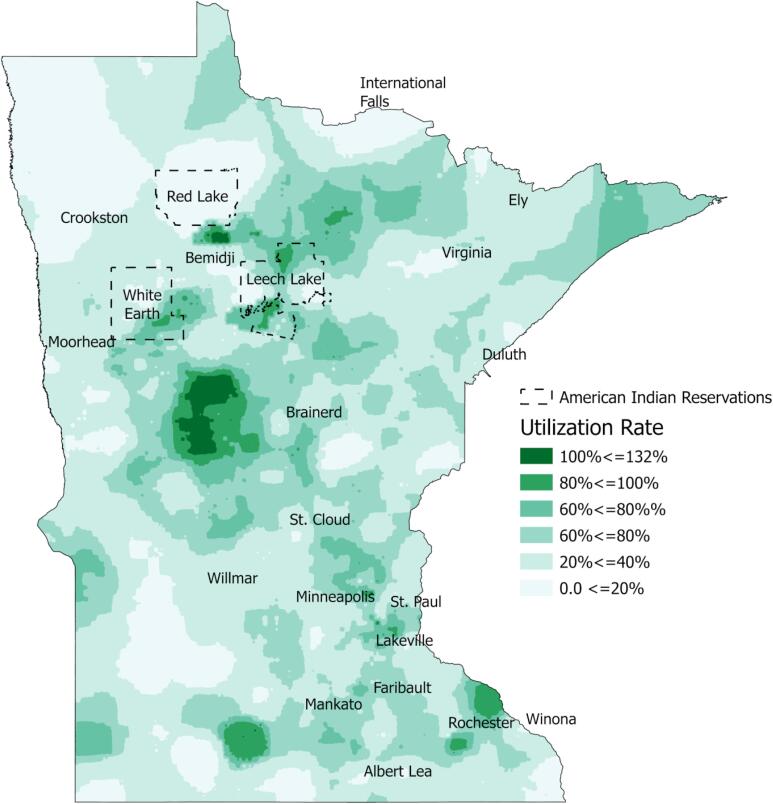
Sage Utilization Rates in Minnesota.

Visual inspection of the map shows clusters of high and low breast cancer screening across the state (Fig. 1). Especially high utilization is found in central Minnesota west of Brainerd, in clusters surrounding Bemidji, and in pockets west of Mankato and adjacent to Rochester. Notably, the clusters of high utilization near Bemidji correspond spatially with the Red Lake, White Earth, and Leech Lake tribal areas. Rochester, MN is the home of the Mayo Clinic, a long standing Sage partner and world class medical clinic.

**Fig. 2:**
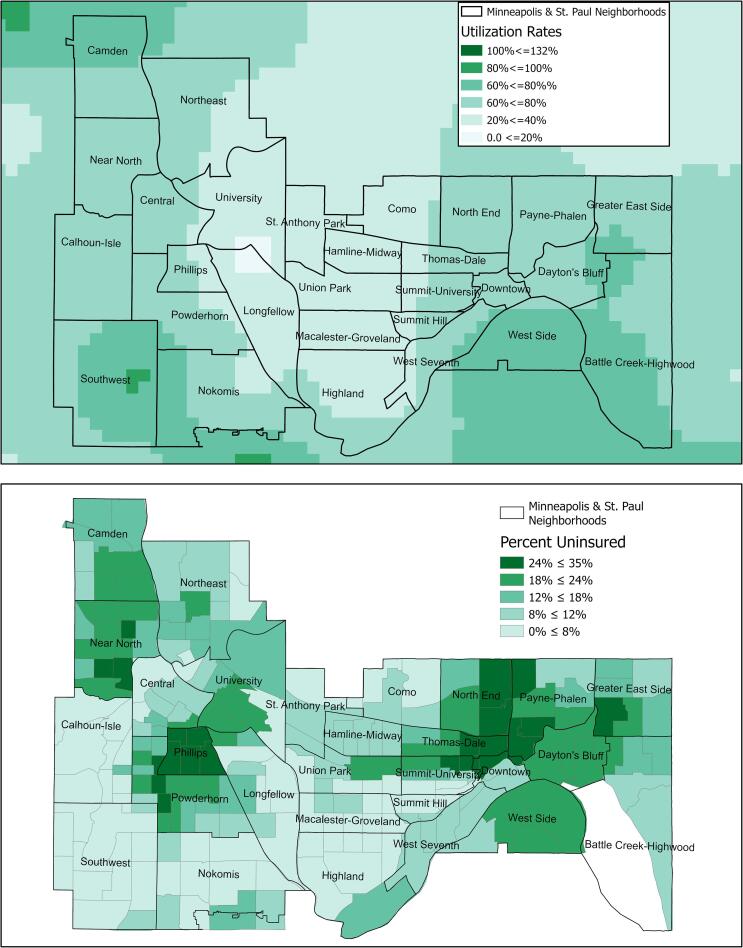
A: Sage utilization rates in Minnesota’s Minneapolis and St. Paul neighborhoods, B: 500 Cities dataset on rates of lack of health insurance in Minneapolis and St. Paul neighborhoods, derived from BRFSS data 2012–2016 (Cebters for Disease Control and Prevention, National Center for Chronic Disease Prevention and Health Promotion, Division of Population Health, 2020). Sage programs can zoom into Minnesota locales and examine breast cancer screening utilization alongside relevant demographic and health data that provide context for Sage’s programmatic efforts, such as rate of lack of health insurance.

Examination of local rates in Minneapolis and St. Paul neighborhoods demonstrate the high-resolutions possible with SAFs (Fig. 2A). Each pixel on the map represents 500 m^2^. The map reveals low utilization in a corridor between the two city centers, and few places where utilization rates are particularly high. Pixels with very high utilization scores in southwest Minneapolis correspond with high income areas with a very small Sage eligible population.

## 4 Discussion

### 4.1 Main findings

Our work adds to the literature through introduction of powerful geospatial methods that quantify the number of individuals eligible for Sage breast cancer screening services and utilization of those services at the local level. We were capable of generating these estimates despite challenges associated with small numbers and a need for local, high-resolution yet reliable estimates. These methods provided the first published state-level and local estimates for NBCCEDP breast screening rates.

Our estimate of statewide utilization of breast cancer screening services (37.2%) is well within the previously reported range of NBCCEDP breast screening rates at the state level (3.2% to 52.8%), yet is higher than previous national estimates of NBCCEDP breast cancer screening utilization that ranged from 10% to 20% for the same time period (Tangka et al., 2006, Howard et al., 2015, Subramanian et al., 2015). Our estimation of Sage’s breast cancer screening is likely higher than previous estimates as we included screenings funding by all revenue streams, not just federal funding, as was the case in previous publications (Tangka et al., 2006, Howard et al., 2015, Subramanian et al., 2015). Our estimated denominator population is similar to previously published denominators; we estimated that 36,979 women 40 and older were eligible for Sage breast screening, while Howard and colleagues estimated that 35,000 women 40 to 64 were eligible for Sage (Howard et al., 2015). Our denominator is likely larger than Howard and colleagues' estimates due to a slightly larger age range and inclusion of underinsured women in the model. These indicators suggest that our new methods reliably and reasonably replicate previous state-level knowledge of NBCCEDP screening uptake in Minnesota. Additionally, Sage programs screened more of the eligible population compared to the median NBCCEDP program, yet all estimates are relatively low and Sage could potentially screen many more women.

The main focus of our paper is the description of internal variation in uptake of Sage breast cancer screening services. Sage breast cancer screening services varied within Minnesota 0% to 100%. Estimates of utilization at the national or state level obscured significant local variation. Local variation in utilization was revealed by SAF methods, and would have remained unknown using common mapping methods that rely on pre-defined administrative units (e.g., ZCTAs). Pre-defined administrative areas are often used in analyses because they are easily accessible. However, a major limitation is that they vary in size and include heterogeneous populations, which can obfuscate health patterns and socially-relevant boundaries within the unit (Galea, 2007). It is important to reveal this variation to document and address both geographic and racial or ethnic disparities in utilization of Sage program resources.

A small percentage of utilization rates range above 100% (0.8% of pixels). These areas reflect spatial filters with 500 or more instances of screening within five years. There are two main explanations for this outcome. First, the breast cancer screening utilization rate is analogous to a standardized incidence ratio, which standardizes for population density. While ratio values generally range from zero to one (i.e., 100%), they can exceed 1 when more cases are observed than expected. Second, some of our model assumptions may underestimate the denominator. It is likely that the RTI Synthesized Dataset, which is based on the 2010 US Census, underestimates the presence of individuals that are undercounted in the census, such as Blacks/African Americans, Hispanics, and American Indians (O’Hare, 2016). In particular, Minnesota is home to a large American Indian population who live on sovereign land and receive screening through American Indian services. The near 100% utilization of Sage breast cancer screening within sovereign American Indian populations can be attributed to both high utilization and some inflation of utilization via denominator underestimation. We also used a constant uninsured and underinsured rate across the entire selected population, which underestimates rates for certain groups. For example, in 2014 the uninsured rate was 26% among Hispanics compared to 12.6% we used in our model (United States Census Bureau. SAHIE, 2017). Additionally, public data on underinsured rates by any demographic feature do not seem to be unavailable. Since we are applying a local spatial model, if the denominator is underestimated due to the RTI dataset or insurance rate assumptions, the utilization rate will be overestimated, resulting in values above > 100%.

This project was enabled by a government-academic partnership. A funded post-doctoral scholar collaborated with state public health to provide mapping expertise for health outcomes. The materials for this project, except for ArcGIS, were open source and free. Code that was used to perform these analyses were shared with the state and available on Github. Therefore, replication of this project is possible, in regards to finance and resources, for other governmental institutions if pursued through collaborative partnerships.

### 4.2 Limitations

A limitation of this research is the accuracy of geocoding of some of the Sage participants. Self-reported addresses can lead to address inaccuracies or inconsistencies that hamper geocoding and rural addresses are known to be more difficult to geocode (Ward et al., 2005). Geolocations determined by ZCTAs and our distribution algorithm could dissipate utilization rates in sparsely populated areas, but this was not tested with a robustness analysis. Our model could be made richer with more detailed uninsured and underinsured information, such as age, race, or income specific rates. This is an area of future work. Due to the scope of the problem, this initial analysis did not determine eligibility and utilization by important demographic variables, Sage recruitment, or previous experience with the Sage program. Factors external to the current project will determine whether explanatory variables can be identified and used to describe why variation occurred. Finally, we limited the analysis of Sage data to 2011 to 2015 to match the available denominator RTI dataset, based on the 2010 US Census. The Minnesota Sage eligible population may have changed since that time, since implementation of the Medicaid expansion under the Affordable Care Act in 2014 (Kaiser Family Foundation, 2019, Levy et al., 2012, Tangka et al., 2020).

## 5 Conclusion

This research demonstrates how spatial approaches, and in particular SAFs, can provide insight into the programmatic uptake of services, in this case utilization rates of breast cancer screening services of a state NBCCEDP. Service uptake can be examined to the neighborhood level to identify areas of great success and neglect. Maps of service utilization can be inspected in the context of maps of relevant contextual variables such as uninsured rates (Fig. 2B), race and ethnicity, or income, providing informative clues as to correlates of service uptake and how to structure successful interventions to increase utilization. In short, the data can be used immediately as a baseline for program evaluation. Statistical analysis of correlates is possible, but beyond the scope of this publication. Finally, local utilization data enhance the capacity of health programs to engage communities, by providing data that are community-specific and relevant (Dubowitz et al., 2011). NBCCEDPs are present in every state and territory in the US and our model can be applied to other NBCCEDPs or similar cancer screening programs, allowing decision-makers the ability to understand screening service utilization at a level of detail previously unavailable.

## CRediT authorship contribution statement

**Kelly D. Hughes:** Conceptualization, Resources, Data curation, Investigation, Writing - original draft. **David Haynes:** Investigation, Methodology, Software, Visualization, Formal analysis, Writing - original draft, Writing - review & editing. **Anne M. Joseph:** Writing - review & editing.

## Declaration of Competing Interest

The authors declare that they have no known competing financial interests or personal relationships that could have appeared to influence the work reported in this paper.
